# 

**Published:** 1891-08

**Authors:** 


CONTENTS:

PAGE

ORIGINAL ARTICLES:
Historical Development of the Horseshoe. By Zippelius........... 363
Age of the Horse, Ox, Dog, and Other Domesticated Animals. By R. S. Huidekoper, M. D., Vet.................... ................ 377
Recent Patents.................................................... 382

EDITORIAL:
United States Veterinary Medical Association....................... 386
Animal Intelligence.............................. ................ 388

SELECTIONS FROM FOREIGN JOURNALS:
German Review...................................................... 389
English Review..................................................... 394

SOCIETY PROCEEDINGS:
Massachusetts Veterinary Medical Society........................... 402

COMMUNICATIONS:
Osteo Porosis. By A. Jasme, V. S............ ....................... 404

REVIEW DEPARTMENT:
A Manual of the Diseases of the Camel, Etc..................... ...... 407
Veterinary Post Mortem Examinations................................ 409
Books and Pamphlets Received....................................... 410

